# Validity and Reliability of the Helkimo Clinical Dysfunction Index for the Diagnosis of Temporomandibular Disorders

**DOI:** 10.3390/diagnostics11030472

**Published:** 2021-03-08

**Authors:** Roger Alonso-Royo, Carmen María Sánchez-Torrelo, Alfonso Javier Ibáñez-Vera, Noelia Zagalaz-Anula, Yolanda Castellote-Caballero, Esteban Obrero-Gaitán, Daniel Rodríguez-Almagro, Rafael Lomas-Vega

**Affiliations:** 1 FisioMedic Clinic, Dos Hermanas, 41701 Sevilla, Spain; rar00032@red.ujaen.es (R.A.-R.); fisiomedic.dh@gmail.com (C.M.S.-T.); 2 Department of Health Sciences, Campus las Lagunillas, University of Jaen, 23071 Jaén, Spain; nzagalaz@ujaen.es (N.Z.-A.); mycastel@ujaen.es (Y.C.-C.); eobrero@ujaen.es (E.O.-G.); dalmagro@ujaen.es (D.R.-A.); rlomas@ujaen.es (R.L.-V.)

**Keywords:** temporomandibular disorder, validity and reliability, questionnaires and survey validity study

## Abstract

The Helkimo Clinical Dysfunction Index (HCDI) is a simple and quick test used to evaluate subjects affected by temporomandibular disorders (TMDs), and its psychometric properties have not been tested. The test evaluates movement, joint function, pain and musculature, providing a quick general overview that could be very useful at different levels of care. For this reason, the aim of this study was to validate the use of the HCDI in a sample of patients with TMD. Methods: The sample consisted of 107 subjects, 60 TMD patients and 47 healthy controls. The study evaluated concurrent validity, inter-rater concordance and predictive values. Results: The HCDI showed moderate to substantial inter-rater concordance among the items and excellent concordance for the total scores. The correlation with other TMD assessment tests was high, the correlation with dizziness was moderate and the correlation with neck pain, headache and overall quality of life was poor. The prediction of TMD showed a sensitivity of 86.67%, a specificity of 68.09% and an area under the curve (AUC) of 0.841. Conclusions: The HCDI is a valid and reliable assessment instrument; its clinimetric properties are adequate, and it has a good ability to discriminate between TMD-affected and TMD-unaffected subjects.

## 1. Introduction

Temporomandibular joint disorders (TMDs) are a very prevalent condition that, according to some authors, are present in 27.4% of adolescents [1] and 25% of adults [2]. Costs in European public hospitals due to erroneous diagnosis of TMD exceed a minimum of €52 and a maximum of €425, with a mean of €146, according to the amounts received from mutual insurance companies and insurers [3]. The analysis of the aetiology of TMDs has focused on several factors such as inflammatory diseases [4], fractures and trauma [5,6], as well as biomedical models related to temporomandibular joints, muscles of mastication and occlusal factors [7]. The management of TMDs includes clinical examination [8] and the use of imaging techniques both for diagnosis and for monitoring the efficacy of treatments [9,10], which classically included the use of botulinum toxin [11], occlusal splint therapy [12] and polyphenols as potential therapeutic agents [13]. TMDs are related to headache, neck pain, shoulder pain, insomnia, vertigo, ocular pain and hearing loss [14], and 91% of TMD patients reported pain, 61.2% joint clicks or crepitation and 53.3% temporomandibular joint limited range of movement [15].

Due to the wide list of related symptoms, diagnostic criteria for temporomandibular disorders (DC/TMDs) were designed for the performance of an exhaustive assessment of each patient [16]; for this reason, an important requirement of time is needed for adequate evaluation with these internationally accepted criteria, which are considered the gold-standard reference test for the diagnosis of temporomandibular disorders. The test examines 12 dimensions that evaluate mandibular movement, type of bite, pain on movement, pain on touch of the musculature, alterations in mandibular movement and headache [16].

According to the cost of misdiagnosis and the time necessary to perform the reference test for TMD diagnosis, it would be beneficial to find a simpler and quicker tool to use as a diagnostic method for TMD in primary care. The Helkimo Clinical Dysfunction Index (HCDI) has been widely used for the clinical diagnosis of TMDs [17,18,19]. It is a simple and quick test that assesses limitations of mandibular movement, pain and joint function. However, the studies that analysed the reliability [20,21] and validity of this tool are old, used a very small sample, applied incorrect statistical techniques and were limited to the analysis of a single clinimetric property [22,23].

Therefore, a thorough analysis of the main properties of the HCDI is necessary, using the DC/TMD protocol as a reference. For this reason, the aim of the study was to assess and test the psychometric properties of the HCDI in patients with TMD.

## 2. Materials and Methods

### 2.1. Participants

To meet the objectives of this work, a cross-sectional validation study was designed. The protocol of this study received the approval of the Research Ethics Committee of Jaén, Spain (date of approval: 27 April 2020; internal code ABR.20/2.TFM). This study was conducted in accordance with the Declaration of Helsinki, good clinical practice guidelines and all applicable laws and regulations, and written informed consent was obtained from all subjects to participate in the study.

The sample size calculation was carried out using the recruitment of at least 10 subjects per item of the scale as a criterion, with a minimum of 80 subjects for validity studies and 20 for reliability [24]. This study was developed between May and August 2020. The sample was selected from the patients of the Dental Medical Center Doctores López Collantes, which provides stomatology services (Dos Hermanas, Sevilla, Spain). and from those at the FisioMedic Clinic (Dos Hermanas, Sevilla, Spain), which provides physiotherapy, general medicine and traumatology services. Recruitment was performed by telephone contact and personal interviews.

### 2.2. Measurements

Once the patients were selected, demographic data were recorded: age, sex, height, weight, body mass index (BMI), educational level, work situation, smoking status, alcoholic habits and physical activity [25].

The diagnostic validity of the HCDI was measured according to the DC/TMD protocol, which is the gold-standard diagnostic test for TMD. The DC/TMD protocol is composed of 12 items that assess muscle and joint pain, pain during jaw movement, headache, bites, noise, obstacles or blockages during jaw movement and discomfort in the palpation of the muscles of the temporomandibular joint. Finally, a diagnostic tree is used to specify a diagnostic result. The DC/TMD protocol has a sensitivity of 86%, a specificity of 98% and an inter-examination reliability of 85% [16].

The main measure was the HCDI. The instrument is comprised of five items, with each assessment having three possible answers, scored as 0, 1 or 5. The first item (A) is related to the limitation in the range of jaw movement and is subdivided into four sections: the maximum opening of the mouth and the protrusion and lateral shift to both sides. In the opening of the mouth, a value of more than 40 mm scores 0 points, a value between 30 and 39 mm scores 1 point and opening less than 30 mm scores 5 points; protrusion and lateral mouth shifts score 0 if the measurement is 7 mm or more, 1 point if the range of motion is between 4 and 6 mm and 5 points if the range is less than 4 mm. These subsections of item A are added together to obtain a subtotal that scores 0 if the sum of the four sections is 0, 1 point if the subtotal is between 1 and 4 points and 5 points if the subtotal is greater than 4 points. The second item (B) evaluates the alterations of joint function that produce deviations, sounds and/or joint locks or blockages; the third item (C) evaluates the presence of pain when performing some movements; the fourth item (D) evaluates muscular pain in the masticatory muscles; and the fifth item (E) evaluates the presence of discomfort or pain in the prearticular area of the temporomandibular joint (TMJ) through palpation. From the sum of the 5 items, we identify no TMJ involvement if the score is 0, mild TMJ involvement when the score ranges from 1 to 9, moderate TMJ involvement if the score ranges between 10 and 19 and severe TMJ involvement for a score between 20 and 25. Previous studies have shown that the HCDI is able to detect TMD-affected subjects with rheumatoid arthritis, with a statistically significant difference between affected and unaffected subjects [26,27,28].

Concurrent validity was also measured with Fonseca’s anamnestic index (FAI), which is made up of 10 questions that can be answered with yes, no or sometimes, and these answers are scored 10, 0 or 5, respectively. This questionnaire classifies patients according to the affectation, with a total score between 0 and 100. The test categorises temporomandibular disorder as not affected when the score is between 0 and 15 points, mild affectation when the score is between 20 and 40 points, moderate affectation when the score is between 45 and 65 points and severe affectation when the score is between 70 and 100 points. The FAI has a Cronbach alpha of 0.826, an intraclass correlation coefficient of 0.937, a cut-off point of >35 points, a sensitivity of 83.33% and a specificity of 77.97% [29,30]. Similarly, the short version of Fonseca’s anamnestic index (SFAI) was also considered; it is a five-question questionnaire that is answered and scored the same as the standard version of the FAI, and the questionnaire categorises patients as unaffected by TMD when the scores is between 0 and 15 points and as affected by TMD when the score is between 20 and 50 points. The SFAI has a sensitivity of 86% and a specificity of 95.5% based on a cut-off point of >17.5 [31].

Pain perception was evaluated by the Numerical Pain-Rating Scale (NPRS) test. The subjects indicate their perceived pain with a number between 0 (no pain) and 10 (the worst pain possible). This tool was used to quantify both the neck and the temporomandibular joint and is the pain assessment test preferred by Spanish-speaking patients. The test has a strong correlation with the Visual Analogue Scale (VAS) and the Four-category Verbal Rating Scale (VRS-4) instruments, with the NPRS being preferred by patients; the Kaiser–Meyer–Olkin (KMO) value is 0.85, with a Bartlett sphericity of <0.01, a landing factor of 0.95 and a lack of implementation percentage of <0.01% [32].

To evaluate the possibility of associated neck disability, the Neck Disability Index test was used; it is a 10-question survey, with answers being reported as a number between 0 and 5. For each question, a score of 0 refers to the total absence of disability, while a score of 5 refers to total disability. In this line, a total score between 0 and 5 indicates absence of disability, 5–14 points indicate low disability, 15–24 point indicates moderate disability and 35–50 points indicate great disability. Cronbach’s alpha is 0.89, and the intraclass coefficient is 0.98, with a Pearson’s correlation coefficient with the visual analogue pain scale of r = 0.65 and with the Northwick Park neck pain questionnaire of r = 0.89 [33].

The presence of vertigo and balance problems was assessed by the Dizziness Handicap Inventory (DHI). This questionnaire is composed of 25 questions that can be answered with yes, no or sometimes, scoring 4, 0 and 2 points, respectively. This questionnaire assesses physical, emotional and functional dimensions, each of which has an independent score in addition to the total score. There is a high correlation between each of the dimensions and the total score (*p* < 0.01); factorial analysis shows a structure formed by three components, and there is perfect correlation with the Dizziness Characteristics and Impact on Quality of Life (UCLA-DQ) (>0.75) [34,35,36].

Headache-associated symptoms were measured with the Headache Impact Test (HIT-6), which is an evaluation questionnaire consisting of six questions that can be answered with usual, almost always, sometimes, rarely and never, with a total score between 36 and 78 points. The correlation between the HIT-6 in different languages is high, it has high reliability, and its items are comparable [37].

Finally, the quality of life was assessed using the 12-item Short-Form Health Survey (SF-12). This questionnaire is the short version of the SF-36 and retains its self-administered form. It results in a Mental Component Summary score and a Physical Component Summary score (PCS-12), differentiating between the two components of the quality of life. The weights of the Spanish version of the SF-12 are similar to those of the original American version, with a correlation of >0.9. The questionnaire explains 91% of the variance of the SF-36 in the sum of the components, and the coefficient of internal consistency is 0.9 for the SF-36 and slightly lower for the SF-12 [38].

### 2.3. Statistical Analysis

Descriptive analysis was performed by calculating means and standard deviations for continuous variables and frequencies and percentages for categorical variables. The Kolmogorov–Smirnov test was used to verify the normality distribution of the continuous variables, and the Levene test was used to test the homoscedasticity of the samples. The confidence level was set at 95% (*p* < 0.05).

To test the agreement between the two raters for the total HCDI score, the intraclass correlation coefficient (ICC) of Shrout and Fleiss was used in a one-way random effects model of the absolute agreement type; it estimates the reliability of single ratings [39]. Reliability was considered poor when the ICC was <0.40, moderate when the ICC was between 0.40 and 0.75, substantial when the ICC was between 0.75 and 0.90 and excellent when the ICC was >0.90. From the ICC, the standard error of measurement (SEM) and the minimum detectable change (MDC) were calculated. The SEM was calculated as the baseline standard deviation (SD) (σbase) minus the square root of (1-Rxx), where Rxx is the ICC. The MDC was quantified at the 95% confidence level (MDC95) from the SEM formula as follows: MDC95 = 1.96 * σbase * “√ (1-ICC), where 1.96 is the z-value corresponding to the 95% confidence interval (MDC95). The MDC provides a good tool for translating the ICC into units of change in the instrument. For measured agreement between two raters for the items, a weighted Kappa coefficient, weighted by quadratic weights, was used [40]. The agreement was considered null if Kappa was <0.00, insignificant if Kappa was between 0.00–0.20, discreet if Kappa was between 0.21–0.40, moderate if Kappa was between 0.41–0.60, substantial if Kappa was between 0.61–0.80 and almost perfect if Kappa was between 0.81–1.00 [41]. In addition, Bland–Altman charts were generated to evaluate the limits of agreement [42].

To analyse the concurrent validity of the HCDI with the FAI, NPRS, NDI, DHI, HIT-6 and SF-12, Pearson’s correlation coefficient r was used. The correlation coefficient was considered strong if it was >0.50 and moderate if it was between 0.30 and 0.50 [43].

The ability of the HCDI to discriminate between TMD patients and healthy subjects was determined using receiver operating characteristic (ROC) curves. First, the classification of the subjects as TMD patients or healthy controls was carried out based on the diagnostic criteria of the DC/TMD protocol, and the total score obtained in the HCDI was evaluated as a variable. In the ROC curve, the fraction of true positives (sensitivity) was represented as a function of the fraction of false positives for different cut-off points. The area under the curve (AUC) was also calculated as a measure of the ability of the score to discriminate between the two diagnostic groups (TMD patients or healthy subjects). The AUC was considered statistically significant when the 95% confidence interval did not include 0.5 [44]. Values between 0.5 and 0.7 indicated low accuracy, values between 0.7 and 0.9 indicated good accuracy and values greater than 0.9 indicated high accuracy [45].

## 3. Results

In all, 158 people were contacted, but the final sample was composed of 107 participants (60 TMD patients and 47 healthy controls), as 51 did not meet the selection criteria or refused to participate. The sociodemographic and anthropometric characteristics of the sample are shown in Table 1.

### 3.1. Inter-Rater Reliability 

**Figure 1:**
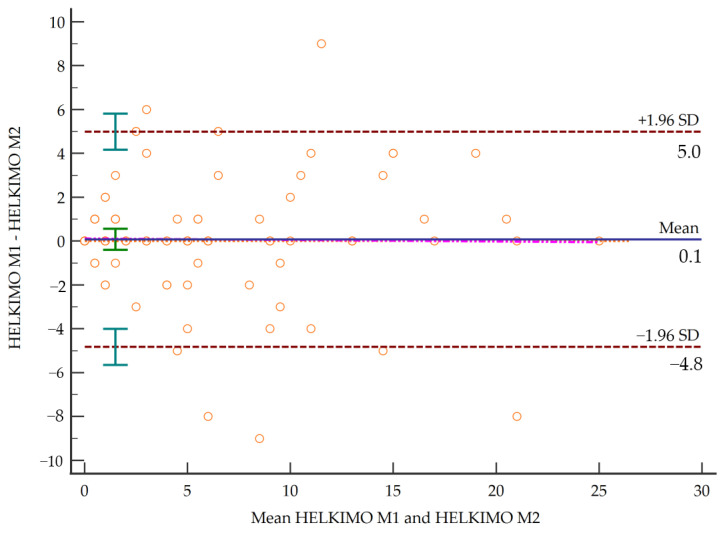
Limits of concordance by Bland–Altman plot.

Results showed a maximum weighted kappa value of 0.774 for item C and a minimum value of 0.426 for item A2. Based on these values, reliability ranged from moderate to substantial, while the total score of the scale reached an excellent degree of concordance of 0.905 (Table 2). Figure 1 shows the Bland–Altman plot. Table 3 shows concurrent validity of the Helkimo Clinical Dysfunction Index with other specific and generic instruments.

### 3.2. Validity and Accuracy of the TMD Diagnostic Ability

**Figure 2:**
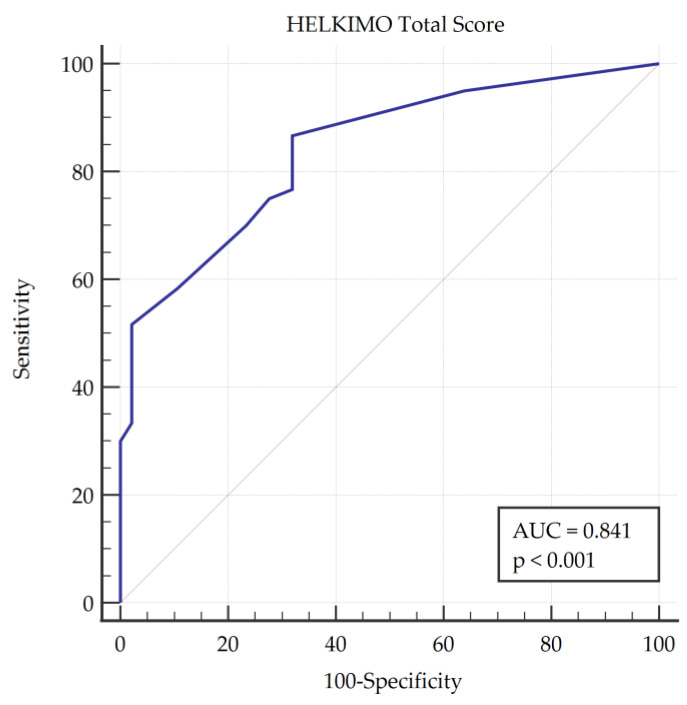
Receiver operating characteristic (ROC) curve plot showing the area under the curve (AUC).

ROC curve analysis found an optimal cut-off point of more than 1 point in the HCDI score that showed a sensitivity of 86.67% with a specificity of 68.09% for the diagnosis of TMDs, making the DC/TMD protocol the gold standard (Table 4). This analysis showed an area under the curve (AUC) of 0.841 (Figure 2), which can be interpreted as good accuracy.

## 4. Discussion

This study evaluated the clinimetric properties of the Helkimo Clinical Dysfunction Index. The data obtained suggested that it is a valid and reliable instrument for evaluating patients with TMD, determining the degree of severity of the condition and discriminating between affected and unaffected patients with TMD. In this study, a total sample of 107 patients was used (60 TMD patients and 47 healthy subjects), and all of them were evaluated by this test, which lasted approximately 4 min. The two groups were comparable, except that a higher proportion of females who suffered from TMD, which is a consistent observation among TMD studies [17,27]. This fact may have led to a reduction in the mean weight and height and a higher proportion of university-educated subjects among the female population [46].

Despite being a commonly used tool for TMD assessment [19], few authors have studied the HCDI in depth. In 1987, Van der Weele et al. conducted an argumentative analysis of the HCDI, studying the pertinence of the construction of such a test to evaluate patients with TMD according to the evidence of the moment. They concluded that there was insufficient scientific evidence to support the use of these items in a diagnostic test for TMD [28]. However, in the analysis of the current scientific evidence regarding the pertinence of the use of these items in a diagnostic test for TMD, there is a general consensus that supports their use, and no evidence casts doubt on it [19,47]. In 2007, Da Cunha et al. conducted a comparative study between the HCDI and the craniomandibular test. As in the present study, they found greater affectation of TMD among women, who represented 70% of the total sample of affected people in the study, and a mean age of 46 years in affected patients, which agrees with the mean age of 43 years observed in this study [27].

Oliveira de Santis et al. conducted the only study analysing the psychometric characteristics of the HCDI and the American Association of Orofacial Pain (AAOP) index in subjects aged between 6 and 18 years, using the DC/TMD protocol as a reference. The authors found a non-statistically significant difference between genders, a sensitivity of 53.40% and a specificity of 77.27% for the HCDI, as well as a low level of accordance between the test being considered and the gold standard [47]. Nonetheless, in the present study, the sensitivity obtained was 86.67%, while the specificity was 68.09%. These differences in the results may be due to the difference in age between samples (46.25 years old in our study, 8.18 years old in the one of Oliveira de Santis et al.), which could indicate that the HCDI is more useful for adults than children.

The present study had some limitations. First, the study sample had a higher proportion of women due to the higher proportion of women affected by TMD. Furthermore, although this study analysed the most common psychometric properties, we did not study the sensitivity to change or the ability to discriminate between different TMD populations. Additionally, this study was carried out on a sample of resident patients in a well-defined geographic location, which limits the generalisation of the results obtained.

## 5. Conclusions

The study shows that the HCDI is suitable for the diagnosis of TMD. The inter-observer concordance was between moderate and substantial for each of the items and excellent for the total score of the test. The HCDI has strong concurrent validity with the FAI, SFAI and NPRS orofacial assessment instruments; moderate validity with the NPRS neck pain assessment, emotional and physical facets and the total DHI value; and poor validity with respect to HIT-6 instruments, the mental and physical components of the SF-12 and the functional component of the DHI. The HCDI shows a sensitivity of 86.67%, a specificity of 68.09% and an AUC of 0.841 to predict the presence of TMD.

## Figures and Tables

**Table 1:** Anthropometric and sociodemographic characteristics of the sample and groups.

		All	*n* = 107	Healthy	*n* = 47	Temporomandibular Disorders (TMDs)	*n* = 60
Weight (kilograms)		72.83	17.05	77.86	19.22	68.90	14.07
Height (meters)		1.63	0.09	1.65	0.09	1.61	0.07
Body mass index		27.48	6.91	28.48	7.10	26.69	6.72
Age (years)		46.25	13.88	49.66	14.56	43.53	12.79
Sex	Female	83	77.6	27	57.45	56	93.3
	Male	24	22.4	20	42.55	4	6.7
Study level	Primary	19	17.8	12	25.53	7	11.7
	Secondary	52	48.6	25	53.19	27	45.0
	University	36	33.6	10	21.28	26	43.3
Physical activity	No	45	42.1	19	40.43	26	43.3
	Yes	62	57.9	28	59.57	34	56.7
Economic level	<€20.000	62	57.9	29	61.70	33	55.0
	>€20.000	45	42.1	18	38.30	27	45.0
Smoker	No	69	64.5	28	59.57	41	68.3
	Yes	13	12.1	6	12.77	7	11.7
	Occasional	12	11.2	6	12.77	6	10.0
	Ex-smoker	13	12.1	7	14.89	6	10.0
Drinker	No	38	35.5	19	40.43	19	31.7
	Regular drinker	6	5.6	3	6.38	3	5.0
	Occasional	63	58.9	25	53.19	38	63.3

**Table 2:** Inter-rater concordance of the Helkimo items and the total score.

Measure	Value	95% Confidence Interval	Degree of Concordance
Item A1	0.62548	0.48243 to 0.76853	Substantial
Item A2	0.42641	0.20367 to 0.64916	Moderate
Item A3	0.51430	0.31876 to 0.70983	Moderate
Item A4	0.64430	0.52330 to 0.76529	Substantial
Item A	0.61987	0.49568 to 0.74407	Substantial
Item B	0.51661	0.37930 to 0.65391	Moderate
Item C	0.77395	0.66415 to 0.88375	Substantial
Item D	0.75750	0.65350 to 0.86149	Substantial
Item E	0.72116	0.60305 to 0.83926	Substantial
Total score	0.9053	0.8642 to 0.9345	Excellent

**Table 3:** Concurrent validity of the Helkimo Clinical Dysfunction Index with other specific and generic instruments.

Variable	Pearson’s r	*p*-Value	Correlation
Fonseca Anamnestic Index	0.692	<0.001	Strong
Short Form of the Fonseca Anamnestic Index	0.626	<0.001	Strong
Numerical Pain-Rating Scale Orofacial	0.777	<0.001	Strong
Numerical Pain-Rating Scale Neck Pain	0.302	0.002	Moderate
Neck Disability Index	0.265	0.006	Poor
Dizziness Handicap Inventory Functional	0.276	0.004	Poor
Dizziness Handicap Inventory Emotional	0.301	0.002	Moderate
Dizziness Handicap Inventory Physical	0.339	<0.001	Moderate
Dizziness Handicap Inventory Total	0.339	<0.001	Moderate
Headache Impact Test 6 items	0.187	0.054	Poor
Physical Component Summary SF-12	0.003	0.975	Poor
Mental Component Summary SF-12	−0.171	0.078	Poor

**Table 4:** Predictive values of the Helkimo Clinical Dysfunction Index (HCDI) total score by ROC curve analysis for the diagnosis of TMDs.

Sensitivity	95% CI	Specificity	95% CI	+LR	95% CI	-LR	95% CI	+PV	95% CI	-PV	95% CI
86.67%	75.4–94.1	68.09%	52.9–80.9	2.72	1.8–4.2	0.20	0.10–0.4	77.6	69.3–84.2	80.0	67.1–88.7

95% CI: 95% confidence interval; +LR: positive likelihood ratio; -LR: negative likelihood ratio; +PV: positive predictive value; -PV: negative predictive value.

## Data Availability

Data available under request to corresponding author due to participants’ consent.

## References

[1] Paduano S., Bucci R., Rongo R., Silva R., Michelotti A. (2020). Prevalence of temporomandibular disorders and oral parafunctions in adolescents from public schools in Southern Italy. Cranio J. Craniomandib. Pract..

[2] Perez C.V., De Leeuw R., Okeson J.P., Carlson C.R., Li H.F., Bush H.M., Falace D.A. (2013). The incidence and prevalence of temporomandibular disorders and posterior open bite in patients receiving mandibular advancement device therapy for obstructive sleep apnea. Sleep Breath.

[3] Nieto Fernández-Pacheco M.J. (2017). Análisis de los Costes Producidos por una Incorrecta Derivación de Pacientes con Síndrome de Disfunción Temporomandibular.

[4] Ibi M. (2019). Inflammation and temporomandibular joint derangement. Biol. Pharm. Bull..

[5] Minervini G., Lucchese A., Perillo L., Serpico R., Minervini G. (2017). Unilateral superior condylar neck fracture with dislocation in a child treated with an acrylic splint in the upper arch for functional repositioning of the mandible. Cranio J. Craniomandib. Pract..

[6] Choi J., Oh N., Kim I.K. (2005). A follow-up study of condyle fracture in children. Int. J. Oral Maxillofac. Surg..

[7] Suvinen T.I., Reade P.C., Kemppainen P., Könönen M., Dworkin S.F. (2005). Review of aetiological concepts of temporomandibular pain disorders: Towards a biopsychosocial model for integration of physical disorder factors with psychological and psychosocial illness impact factors. Eur. J. Pain.

[8] Fernández-de-las-Peñas C., Von Piekartz H. (2020). Clinical reasoning for the examination and physical therapy treatment of temporomandibular disorders (TMD): A narrative literature review. J. Clin. Med..

[9] Eberhard D. (2002). The efficacy of anterior repositioning splint therapy studied by magnetic resonance imaging. Eur. J. Orthod..

[10] Minervini G., Nucci L., Lanza A., Femiano F., Contaldo M., Grassia V. (2020). Temporomandibular disc displacement with reduction treated with anterior repositioning splint: A 2-year clinical and magnetic resonance imaging (MRI) follow-up. J. Biol. Regul. Homeost. Agents.

[11] Canter H.I., Kayikcioglu A., Aksu M., Mavili M.E. (2007). Botulinum toxin in closed treatment of mandibular condylar fracture. Ann. Plast. Surg..

[12] Fayed M., El-Mangoury N., El-Bpkle D., Belal A. (2004). Occlusal splint therapy and magnetic resonance imaging. Worlf J. Orthod..

[13] Moccia S., Nucci L., Spagnuolo C., d’Apuzzo F., Piancino M.G., Minervini G. (2020). Polyphenols as potential agents in the management of temporomandibular disorders. Appl. Sci..

[14] Ting J., Li J., Zhen Kang S. (2005). A primary research on the concomitant syntoms of temporomandibular joint pain. Zhonghua Kou Qiang Yi Xue Za Zhi.

[15] Katsoulis K., Bassetti R., Windecker Getaz I., Mericske Stern R., Katsoulis J. (2012). Temporomandibular disorders/myoarthropathy of the masticatory system. Res. Sci..

[16] Schiffman E., Ohrbach R., Truelove E., Look J., Anderson G., Goulet J.-P.P., List T., Svensson P., Gonzalez Y., Lobbezoo F. (2014). Diagnostic criteria for temporomandibular disorders (DC/TMD) for clinical and research applications: Recommendations of the International RDC/TMD Consortium Network and Orofacial Pain Special Interest Group. J. Oral Facial Pain Headache.

[17] Rani S., Pawah S., Gola S., Bakshi M. (2017). Analysis of Helkimo index for temporomandibular disorder diagnosis in the dental students of Faridabad city: A cross-sectional study. J. Indian Prosthodont. Soc..

[18] Suhas S., Ramdas S., Lingam P., Naveen Kumar H., Sasidharan A., Aadithya R. (2017). Assessment of temporomandibular joint dysfunction in condylar fracture of the mandible using the Helkimo index. Indian J. Plast. Surg..

[19] Nokar S., Sadighpour L., Shirzad H., Shahrokhi Rad A., Keshvad A. (2019). Evaluation of signs, symptoms, and occlusal factors among patients with temporomandibular disorders according to Helkimo index. Cranio J. Craniomandib. Pract..

[20] Fu K., Ma X., Zhang Z., Tian Y., Zhou Y., Zhao Y. (2002). Study on the use of temporomandibular joint dysfunction index in temporomandibular disorders-PubMed. Zhonghua Kou Qiang Yi Xue Za Zhi.

[21] John M., Zwijnenburg A. (2001). Interobserver variability in assessment of signs of TMD-PubMed. Int. J. Prosthodont..

[22] Abud M.C., Figueiredo M.D., dos Santos M.B.F., Consani R.L.X., Marchini L. (2011). Correlation of prosthetic status with the GOHAI and TMD indices-PubMed. Eur. J. Prosthodont. Rest. Dent..

[23] Pocock P.R., Mamandras A.H., Bellamy N. (1992). Evaluation of an anamnestic questionnaire as an instrument for investigating potential relationships between orthodontic therapy and temporomandibular disorders. Am. J. Orthod. Dentofac. Orthop..

[24] Hobart J.C., Cano S.J., Warner T.T., Thompson A.J. (2012). What sample sizes for reliability and validity studies in neurology?. J. Neurol..

[25] World Health Organization (2013). 2013–2020 Global Action Plan for the Prevention and Control of Noncommunicable Diseases.

[26] Duinkerke A.S.H., Luteijn F., Bouman T.K., Jong H.P. (1986). Reproducibility of a palpation test for the stomatognathic system. Community Dent. Oral Epidemiol..

[27] Da Cunha S.C., Nogueira R.V.B., Duarte Â.P., Vasconcelos B.C.D.E., Almeida R.D.A.C. (2007). Análise dos índices de Helkimo e craniomandibular para diagnóstico de desordens temporomandibulares em pacientes com artrite reumatóide. Braz. J. Otorhinolaryngol..

[28] van der Weele L.T., Dibbets J.M.H. (1987). Helkimo’s index: A scale or just a set of symptoms?. J. Oral Rehabil..

[29] Rodrigues-Bigaton D., de Castro E.M., Pires P.F. (2017). Factor and Rasch analysis of the Fonseca anamnestic index for the diagnosis of myogenous temporomandibular disorder. Braz. J. Phys. Ther..

[30] Sánchez-Torrelo C., Zagalaz-Anula N., Alonso-Royo R., Ibáñez-Vera A., López-Collantes J., Rodríguez-Almagro D., Obrero-Gaitán E., Lomas-Vega R. (2020). Transcultural adaptation and validation of the Fonseca Anamnestic Index in a Spanish population with temporomandibular disorders. J. Clin. Med..

[31] Pires P.F., de Castro E.M., Pelai E.B., de Arruda A.B.C., Rodrigues-Bigaton D. (2018). Analysis of the accuracy and reliability of the Short-Form Fonseca Anamnestic Index in the diagnosis of myogenous temporomandibular disorder in women. Braz. J. Phys. Ther..

[32] Jensen M.P., Castarlenas E., Roy R., Tomé Pires C., Racine M., Pathak A., Miró J. (2019). The utility and construct validity of four measures of pain intensity: Results from a University-Based Study in Spain. Pain Med..

[33] Andrade Ortega J.A., Delgado Martínez A.D., Almécija Ruiz R. (2010). Validation of a Spanish version of the Neck Disability Index. Spine.

[34] Jacobson G.P., Newman C.W. (1990). The Development of the Dizziness Handicap Inventory. Arch. Otolaryngol. Head Neck Surg..

[35] Perez N., Garmendia I., García-Granero M., Martin E., García-Tapia R. (2001). Factor analysis and correlation between Dizziness Handicap Inventory and Dizziness Characteristics and Impact on Quality of Life Scales. Acta Oto-Laryngol. Suppl..

[36] Pérez N., Garmendia I., Martín E., García-Tapia R. (2000). Cultural adaptation of 2 questionnaires for health measurements in patients with vertigo. Acta Otorrinolaringol. Esp..

[37] Martin M., Blaisdell B., Kwong J.W., Bjorner J.B. (2004). The Short-Form Headache Impact Test (HIT-6) was psychometrically equivalent in nine languages. J. Clin. Epidemiol..

[38] Vilagut G., Valderas J.M., Ferrer M., Garin O., López-García E., Alonso J. (2008). Interpretation of SF-36 and SF-12 questionnaires in Spain: Physical and mental components. Med. Clin..

[39] Shrout P.E., Fleiss J.L. (1979). Intraclass correlations: Uses in assessing rater reliability. Psychol. Bull..

[40] Brenner H., Kliebsch U. (1996). Dependence of weighted kappa coefficients on the number of categories. Epidemiology.

[41] Landis J., Koch G.G. (1977). The measurement of the observer agreement for categorial data. Biometrics.

[42] Bland J., Altman D.G. (1999). Measuring agreement in method comparison studies. Stat. Methods Med. Res..

[43] Cohen J., Hillsdale N.J. (1998). Statistical Power Analysis for the Behavioral Sciencies.

[44] Zweig M.H., Campbell G. (1993). Receiver-operating characteristic (ROC) plots: A fundamental evaluation tool in clinical medicine. Clin. Chem..

[45] Swets J. (1988). Measuring the accuracy of diagnostic information. Science.

[46] Ministerio de Ciencia e Innovación de España Estadística de Estudiantes. https://www.ciencia.gob.es/portal/site/MICINN/menuitem.7eeac5cd345b4f34f09dfd1001432ea0/?vgnextoid=0930dd449de8b610VgnVCM1000001d04140aRCRD.

[47] de Santis T.O., Motta L.J., Biasotto-Gonzalez D.A., Mesquita-Ferrari R.A., Fernandes K.P.S., de Godoy C.H.L., Alfaya T.A., Bussadori S.K. (2014). Accuracy study of the main screening tools for temporomandibular disorder in children and adolescents. J. Bodyw. Mov. Ther..

